# “Lazarus Response” When Feto-Maternal Microchimerism Kicks in: Spontaneous Remission in Refractory Primary Mediastinal B Cell Lymphoma Following Twin Pregnancy

**DOI:** 10.3390/diagnostics14182084

**Published:** 2024-09-20

**Authors:** Radu Andrei Tomai, Sabina Iluta, Adrian Bogdan Tigu, Madalina Nistor, Anamaria Bancos, Diana Cenariu, Ciprian Jitaru, Sergiu Patcas, Delia Dima, David Kegyes, Sanda Buruiana, Mihnea Zdrenghea, Alina Daniela Tanase, Ciprian Tomuleasa, Romeo Micu

**Affiliations:** 1 Department of Haematology, Ion Chiricuta Institute of Oncology, 400015 Cluj-Napoca, Romania; raduandreitomai@gmail.com (R.A.T.); iluta.sabina@yahoo.com (S.I.); ani_maribancos@yahoo.com (A.B.); ciprianjitaru.jitaru@gmail.com (C.J.); deli_dima@yahoo.com (D.D.); m.zdrenghea@yahoo.com (M.Z.); 2 Department of Translational Medicine, Institute of Medical Research and Life Sciences—MEDFUTURE, “Iuliu Hațieganu” University of Medicine and Pharmacy, 400337 Cluj-Napoca, Romania; tiguadrianbogdan@yahoo.com (A.B.T.); diacenariu@gmail.com (D.C.); kegyesdavid70@gmail.com (D.K.); 3 Department of Hematology, Iuliu Hațieganu University of Medicine and Pharmacy, 400337 Cluj-Napoca, Romania; 4 Department of Obstetrics and Gynecology, Iuliu Hațieganu University of Medicine and Pharmacy, 400337 Cluj-Napoca, Romania; spastacas@yahoo.com (S.P.); romeomicu@hotmail.com (R.M.); 5 Department of Hematology, Nicolae Testemitanu University of Medicine and Pharmacy, MD-2004 Chisinau, Moldova; sanda.buruiana@usmf.md; 6 Department of Stem Cell Transplantation, Fundeni Clinical Institute, 022338 Bucharest, Romania; alinadanielatanase@yahoo.com

**Keywords:** microchimerism, SRY, remission, gene analysis

## Abstract

**Background**: Spontaneous remission of cancer is a rare and poorly understood phenomenon characterized by complete or partial remission of a malignancy in the absence of or with inadequate treatment. The underlying mechanism for such occurrences is poorly understood, however, immune mechanisms seem to play an important role in such cases. In recent years increasingly more data have become available in favor of the clinical benefit of low levels of chimerism in hematologic malignancies. One such instance of naturally occurring low-level chimerism is feto-maternal microchimerism which has been shown to influence cancer progression and, in some instances, to be a protective factor against malignancy. **Case report**: We report a case of a young female patient with aggressive primary mediastinal large B cell lymphoma refractory to two lines of chemo-immunotherapy achieving sustained complete metabolic remission of tumor while pregnant with twins. **Results**: A focus on feto-maternal microchimerism during and after pregnancy revealed transient levels of feto-maternal microchimerism in the peripheral blood of the patient as measured by quantifying the Y-chromosome-linked SRY gene. **Conclusions**: Microchimerism presents significant potential for enhancing our comprehension of disease mechanisms, uncovering novel therapeutic targets, and refining diagnostic and treatment approaches, especially concerning cancer.

## 1. Background on Materno-Fetal Microchimerism

Spontaneous remission (SR) of malignancies is a poorly understood and rare phenomenon characterized by complete or partial remission of a malignancy without any treatment or with inadequate treatment considered insufficient to induce remission of tumor [1]. This phenomenon has been described in various malignancies such as malignant melanoma, neuroblastoma, bladder and kidney cancer, Kaposi sarcomas and leukemias [2,3]. This term, spontaneous remission, is rather a misnomer as such tumor remissions are idiopathic, but increasingly more data are available supporting an immunomodulatory mechanism for tumor regression in the absence of chemotherapy, including the effectiveness of novel immune therapies [1,4].

Immune modulation has proved to be an effective approach to treatment of hematologic malignancies, with one example being the use of donor lymphocyte infusions in relapsing patients following allogeneic stem cell transplantation resulting in disease remission. Recent advances have proved that even transient or persistent low levels of chimerism (<1%), as in the case of microtransplants, are effective in improving complete remission rates in acute myeloid leukemia, opening a path towards new approaches for treatment based on allogeneic immunity [5].

One instance of naturally occurring persistent low-level chimerism is fetal–maternal microchimerism which is defined by the presence of genetically distinct cells originating from a fetus, that can be found in the blood and organs of the mother as a result of transplacental trafficking [6]. These cells can sometimes be found in the mother’s tissues for decades without apparent graft-related incompatibilities [7].

Evidence that the percentage of male microchimerism in some cancer patients is significantly lower than in normal volunteers raises the question of the role of fetal–maternal microchimerism in oncogenesis and cancer protection, however, currently available data are insufficient to clarify this issue [6].

With inherent plasticity, fetal cells are considered equivalent to stem cells and pass into maternal circulation through bidirectional trafficking. This is believed to happen in pregnancy, before the formation of the placenta [8]. These cells migrate into tissues and adopt tissue specific phenotypes, with proven differentiation into ectodermal, endodermal, mesodermal and trophectodermal tissues [9]. Significantly high prevalence of fetal origin has been reported in T and B lymphocyte populations and natural killer and antigen-presenting cells [10].

The data available for fetal–maternal microchimerism are mostly based on male fetal microchimerism due to the accessibility of using the Y chromosome or Y-chromosome-encoded genes as a surrogate for the presence of fetal cells in the mother [11].

Initial reports have shown lower prevalence of fetal–maternal microchimerism in breast cancer patients compared to normal controls [12]. These findings raised hopes regarding the potential immune-modulating effects of fetal microchimeric cells (FMCs) and further data proposing a protective effect against hematologic, papillary thyroid cancers, glioblastoma and meningioma supported this hypothesis, however, further reports have shown conflicting data, with fourfold increased risk of colon cancer in females with FMCs [10,11]. Additionally, effects on tumor progression have been reported for thyroid and uterine cancer, where FMCs have been associated with less extensive or more aggressive cancers, respectively. Remarkably, different prevalences of FMCs have been shown in solid and hematologic cancers, with higher frequency of FMCs in the latter [10,13]. The heterogeneous effects on oncogenesis and protection against cancer might be related to FMC transdifferentiation and their role at tumor sites. Particularly, fetal cells with hematopoietic differentiation have been postulated to have a protective role in some cancers, relating to immune surveillance and tumor destruction in a manner similar to that observed in the case of allogeneic stem cell transplantation [10,12].

With hope of providing additional information towards bridging this gap, we present the case of a female patient with double refractory primary mediastinal diffuse large B cell lymphoma attaining sustained spontaneous remission while pregnant with twins and associated with male microchimerism.

## 2. Case Report

A 27-year-old nulliparous female with a medical history of polycystic ovary syndrome and obesity presented to the doctor for progressive fatigue and shortness of breath with an onset two months prior and recent aggravation. She was referred to the pneumology department for evaluation. The clinical examination revealed significant dyspnea, and history revealed onset of night sweats, fatigue and unintentional weight loss of 10 kg within the last 2 months. This prompted a chest CT scan evaluation which revealed a mediastinal mass. Mediastinotomy and tumor biopsy were performed, and the preliminary pathology report suggested malignant lymphoproliferation. She was thus transferred to the hematology unit for further investigations for suspected lymphoma.

Upon examination the clinical findings showed generalized pallor, grade II obesity and diminished right vesicular breath sounds. There were no palpable lymph nodes and no organomegaly. The surgical scar was clean and heart auscultation was normal. Blood pressure was 110/70 mHg and SpO2 88%. Her laboratory values showed abnormalities, with microcytic anemia, Hb 8.2 g/dL, elevated LDH of 608 IU/L, alkaline phosphatase of 460 IU/L and a spontaneous INR of 1.57.

**Figure 1:**
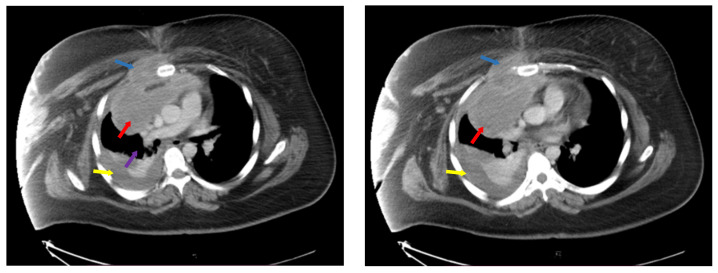
Computer tomography at diagnosis showing mediastinal adenopathy block (red arrows), with compression of vasculature (purple arrow) and invasion of thoracic wall and right pectoral muscle (blue arrows), pleural effusion (yellow arrows). Left side—ce reprezinta (descriere), Right side—ce reprezinta (descriere).

Disease extension was evaluated with neck, chest, abdomen and pelvis computer tomography (CT) as seen in Figure 1., which revealed multiple retroclavicular adenopathies, with compression of right subclavian vein, bilateral axillary and cervical adenopathies of up to 19 mm and an anterior mediastinal adenopathy block of 110/85 mm, with invasion of anterior thoracic wall and right pectoris muscle, inclusion and compression of thoracic aorta and superior vena cava. There was a right pleural effusion of 25 mm and pericardial effusion of 10 mm.

The pathology report showed a malignant neoplasia composed of medium to large pleomorphic cells and important tissue necrosis. Immunohistochemistry showed the cells were positive for CD20 and Pax 5, with heterogenous positivity for CD10, Bcl-6, MUM1 and CD30 while CD15, CD3, TdT staining was negative. The Ki-67 proliferation index was 80%. A formal diagnosis of stage II of primary mediastinal B cell lymphoma was made, with an IPI score of 2. Infusional chemoimmunotherapy was opted for as a first line of treatment and she was treated according to the rituximab, dose-adjusted etoposide, prednisone, vincristine, cyclophosphamide and doxorubicin (R-DA-EPOCH) regimen.

After the 3rd cycle of chemoimmunotherapy, she developed a central line methicillin-resistant Staphylococcus epidermidis (MRSE) infection which required catheter suppression and resolved under antibiotic therapy with vancomycin. Due to obesity and the large mediastinal mass, central venous catheter placement was difficult and traumatic for the patient. Following the infectious episode, the patient firmly refused placement of a new central line, therefore infusion chemotherapy was no longer possible and, instead, treatment was pursued with the rituximab, cyclophosphamide, doxorubicin, vincristine, prednisone, etoposide (R-CHOEP) regimen as an alternative with easier administration via a peripheral intravenous line.

Response to treatment was assessed by positron emission tomography (PET-CT) after six cycles of chemoimmunotherapy (three R-DA-EPOCH and three R-CHOP-Etoposide). The examination showed persistent disease with a supraclavicular adenopathy of 41 mm with a standardized uptake value (SUV) of 6.30, a right anterior mediastinal paratracheal mass of 56 mm, with paracardial extension of 83/59 mm, invasion of right thoracic wall and right pectoris muscle, central necrosis and an SUV of 7.53. There were no hypermetabolic axillary lymph nodes and the pericardial effusion had remitted while the right pleural effusion had diminished. Hepatic SUV was 1.64 and mediastinal SUV was 1.35. The Deauville score was 5.

The patient was started on second line treatment with rituximab, ifosfamide, carboplatin (R-ICE) and response to treatment was revaluated after the 3rd cycle of chemotherapy. Interim PET-CT showed further progression of disease with right supraclavicular mass of 84/43 mm with mediastinal paratracheal extension, SUV of 6.52 in the supraclavicular region and 5.58 in the paratracheal region. There was a right hilar mass of 53/56 mm with central necrosis and increased SUV of 10.31 accompanied by right middle lobe atelectasis. Additionally, a new hypermetabolic lesion of 7 mm was identified at the rectosigmoid junction. Hepatic SUV was 1.79 and mediastinal SUV was 1.25. The Deauville score was 5.

A third line of treatment for double refractory disease was scheduled, however, the patient did not show up for further treatment of evaluation until 5 months later. Upon evaluation she was discovered to be in the 6th week of amenorrhea and subsequently confirmed pregnant with twins. A therapeutic pregnancy termination was proposed in order to further pursue chemotherapy for her lymphoma; however, she refused and was once again lost to follow-up. She eventually returned for revaluation 6 months later, in the 34th week of pregnancy, in seemingly good overall condition. Disease extension was assessed by thorax MRI which showed morphologic remission of tumor mass, with right mediastinal, paracardial encapsulated mass of 34/18 mm with central necrosis and minimal thoracic wall invasion. No additional thoracic or mediastinal masses and no vascular com-pression were identified. There were no axillary, supraclavicular or cervical adenopathies and no pleural or pericardial collections. In this setting, a watch and wait approach was taken and the patient was revaluated with a PET-CT at 3 months after giving birth to two healthy male twins. This examination confirmed the previous findings, with a 24 mm residual mass with central necrosis and calcifications, with a normal SUV of 1.53 and passive adjacent focal atelectasis within the right middle lung lobe. No residual metabolically active adenopathies were identified. Hepatic and mediastinal SUVs were 1.93 and 1.44, respectively, and Deauville score was 3, suggesting complete metabolic remission of lymphoma.

Long-term follow-up showed persistence of remission, up to 4 years later, with a PET-CT showing persistence of the residual mass with calcifications and central necrosis, now measuring 25 mm and with a lower SUV of 1.17, with reference hepatic and mediastinal SUVs of 1.63 and 1.43, respectively, and Deaville score of 2.

## 3. Proving Materno-Fetal Microchimerism

**Figure 2:**
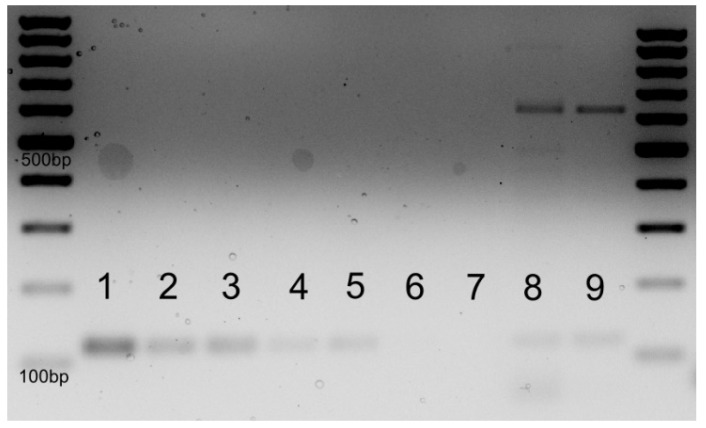
Electrophoresis for gDNA amplification of SRY. The SRY amplicon is 113 bp in length, and GAPDH is 89 bp in length. Male gDNA 100 ng—1; 75 ng—2; 50 ng—3; 25 ng—4; 10 ng—5; 1 ng—6; Female negative control gDNA—7; Patient DNA during pregnancy week 34 gDNA—8; patient DNA 4 years later—9.

We hypothesized that the spontaneous remission of lymphoma in this case might be attributable an influence of feto-maternal microchimerism. To support this hypothesis, we extracted genomic DNA (gDNA) from the peripheral blood of the mother and used the gDNA template to detect the presence of the Y chromosome. We used the SRY gene located on the Y chromosome as a surrogate for the presence of male feto-maternal microchimerism and selected housekeeping gene GAPDH as a positive control for DNA amplification. For positive and negative control samples for the SRY gene, we used DNA extracted from peripheral blood mononucleated cells of an adult male and adult nulliparous female volunteers. DNA samples of the patient collected at two different time points, during the 34th week of pregnancy and 4 years later, were analyzed. We will further refer to these DNA samples as patient sample T1 and patient sample T2, respectively. We performed PCR for the selected genes followed by agarose gel electrophoresis and RT PCR quantification of the SRY template. The electrophoresis showed SRY amplification in DNA samples of the patient as well as the positive control sample. There was no amplification of SRY in the negative control sample. Technique sensitivity for PCR followed by electrophoresis was assessed by determining the lower limit of detection for the SRY gene by PCR, thus several dilutions of the male gDNA were used, starting from 100 ng integral male DNA, diluted to 75%, 50%, 25%, 10% and 1% male gDNA in the sample. As depicted in Figure 2, the limit of detection for the SRY amplicon was between 10% and 1% gDNA. Technique specificity is dependent on the SyBr Green kit and manufacturer and is regarded as high when the protocol is followed, with the use of negative controls to test for possible contamination. The patient gDNA samples displayed amplification bands for SRY and GAPDH, while the negative control had no amplification of SRY. The gDNA was extracted using a PureLink DNA extraction Kit (Invitrogen, Waltham, CA, USA). The primers were designed and ordered from Biolegio (Nijmegen, the Netherlands), and the sequences are as follows: SRY forward primer GGC AAC GTC GTC CAG GAT AGA GTG A and SRY reverse primer TGC TGA TCT CTG AGT TTC GCA TT, GAPDH forward primer AGC CAC ATC GCT CAG ACA C and GAPDH reverse primer GCC CAA TAC GAC CAA ATC C. PCR plates, optical foils, SyBr Green Master Mix 2X and ultrapure water were purchased from Applied Biosystems (Waltham, CA, USA).

PCR amplification as shown by electrophoresis provided weak signals, thus we decided to perform an RT PCR assay, using the same gDNA templates and primers. RT PCR was performed using SyBr Green Master Mix. A standard curve for SRY and GAPDH amplification was generated starting from the positive control sample, with 50 ng male gDNA, diluting the samples in ratios of 1:2, 1:4, 1:8 and 1:16. This allowed the generation of positive standard curves for both SRY and GAPDH with good slope and respecting the two-fold dilution.

The RT PCR results showed SRY gene amplification solely in patient sample T1, while patient sample T2 was negative. This was verified against positive and negative control samples for SRY with male gDNA and negative nulliparous female gDNA, respectively, and against the internal amplification control of the GAPDH gene. These results indicate that the presence of SRY was detected only at the 34-week pregnancy time point and was not detectable 4 years later.

**Figure 3:**
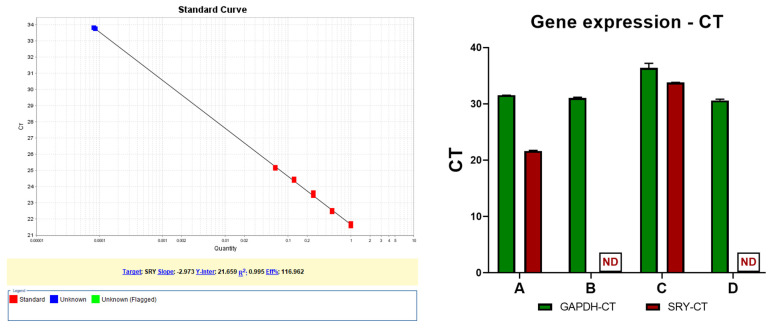
SRY gene DNA quantification in gDNA via RT PCR. The red dots are the standard diluted samples, starting from 50 ng gDNA. The blue dots are the two positive SRY samples from patient in duplicate samples. A—Male control sample (50 ng/reaction); B—Female control sample (50 ng/reaction), C—Patient at 34 weeks of pregnancy (50 ng/reaction), D—Patient after 4 years (50 ng/reaction), ND—not detected. Left side—image depicting calibration curve and linearity for SRY determination, Right side—graphic depicting the comparison between gene amplification for SRY and housekeeping gene GAPDH (with the maximum determination at cycle 40).

The standard curve was performed by designating the 50 ng gDNA as concentration 1, then the serial dilutions were 0.5, 0.25, 0.125, and 0.0625. By using the calculated slope of −2.9728, the Applied Biosystems software on a Step OnePlus RT PCR Machine calculated the SRY quantity from the patient sample T1 at 8 × 10^−5^ (0.00008). With a reference value of 50 ng DNA corresponding to concentration 1, the patient sample T1 is calculated as having 0.004 ng SRY product, as shown in Figure 3—left. The CT values highlight the presence of SRY and GAPDH in the DNA, as shown in Figure 3—right.

Amplification data showed SRY amplification at cycle 21 in the positive control sample with concentration 1 and at cycle 26 at concentration 0.0625. The SRY amplicon in patient sample T1 was detected at cycle 33.8 which indicates that the amount of SRY product is lower compared to the control. No SRY product was detected in patient sample T2 after 40 cycles or in the negative control. All samples had detectable GAPDH product between cycles 31 and 36, with the standard curve containing points detected at cycle 31 and up to 35.

## 4. Discussion

Recent advances in the treatment of hematologic malignancies, with the advent of CAR-T therapies and effective immune therapies, have shown impressive results as well as significantly lower toxicities as compared to chemotherapy. These new encouraging directions point to immune therapies as the future of cancer treatment [14]. The effectiveness of CAR-T therapy in treatment of hematologic malignancies is an argument for a new approach of harnessing the body’s own immune system in the fight against cancer.

The potentially protective role of fetal cells and their function in immune surveil-lance against cancer makes for an intriguing hypothesis regarding the potential applications of this phenomenon in immune therapies. Supporting arguments for this hypothesis come from the field of hematopoietic stem cell transplantation where cord blood transplants are effective in the consolidation of response in acute myeloid leukemia [15,16].

In haplo-cord stem cell transplantation, combining unrelated cord blood with a haplo-identical donor graft, higher cord blood chimerism was associated with lesser rates of disease relapse and higher overall survival, whereas higher degrees of haplo-identical donor chimerism lead to more rapid immune reconstitution. This suggests an important graft versus leukemia effect of the cord blood graft which is advantageous towards disease control [15].

A rather novel approach in the field of stem cell transplantation is making use of the graft versus leukemia (GVL) effect and that is micro stem cell transplantation (MST). This approach relies on infusion of G-CSF mobilized peripheral blood mononucleates in patients following standard induction and consolidation chemotherapy for acute myeloid leukemia, however, with no immunosuppressive treatment. The objective of MST is obtaining transient or durable microchimerism with the aim of controlling hematologic malignancy through the GVL effect. Several studies have shown encouraging results, with benefits of remission rates and relapse-free survival over chemotherapy alone, with limited toxicity [5,17]. Importantly, HLA mismatch might lead to better efficacy of MST with no additional toxicity [18].

The impact of fetal–maternal microchimerism, as well as its role in cancer protection and oncogenesis, remains unclear, however, the complete metabolic remission of the double refractory primary mediastinal B cell lymphoma during pregnancy in the case presented raises an important question of causality between FMCs and tumor remission. We have demonstrated by PCR the presence of very low levels of the SRY gene in the peripheral blood of the mother as a surrogate for male FMCs, supporting this hypothesis. The low levels of FMCs detected require validation by alternative techniques, however, this indicates the potential tumor-modulating effects of low levels of allogeneic circulating cells. The detection and quantification of the SRY gene by qPCR during pregnancy but negativity in the follow-up sample at the 4-year time point confirms the higher levels of fetal–maternal microchimerism during pregnancy as well as the potential transient aspect of FMCs.

Further investigation into the mechanism and influence of fetal–maternal microchimerism is required to correctly articulate potential clinical applications for this phenomenon. In vitro and animal models are feasible and replicable scenarios in which to analyze and characterize the fetal–maternal chimerism mechanisms and its influence on cancer. While this research could be extended to haplo-identical microchimerism of different origins, it might be the uniqueness of maternal tolerance towards fetal antigens which allows for the delicate balance between graft versus tumor and lack of host versus graft effect in patients who are not immune suppressed by lymphodepletion or myeloablation. Thus, another important direction for research would be for ways in which to extend this immune tolerance for allogeneic cells to hosts who do not share a mother–child bond with the donor.

Attaining an effective graft versus tumor effect with limited graft versus host toxicity and, furthermore, with limited need for immune suppression is a holy grail in the field of hematopoietic transplantation, but FMCs might just present all these features albeit to a lower extent given the low chimerism. An antitumoral effect of FMCs would respond to the need for low-intensity effective therapies for frail and refractory patients. Additionally, a well-tolerated therapy would perhaps show a benefit for consolidative therapy in lymphoma patients as well as in myeloid malignancies.

Future research perspectives in this field focus on developing more accurate and reproducible techniques for assessing fetal–maternal microchimerism, characterizing the origins of FMCs and distinguishing differentiation patterns of FMCs and functions relating to distribution in various tumors, organs, wounds and circulating blood and implications in the development of autoimmune disorders [10,19]. Efforts focused on characterizing the cancer-like properties of FMCs such as immune evasion aim at clarifying the risks posed by these naturally occurring cells, which would further translate into risks associated with FMC-derived therapies [20].

An important aspect which needs to be taken into consideration in the case of this patient is the influence of pregnancy-associated hormone changes on lymphoma. It has been reported that increased exposure to estrogens and progesterone reduces lymphopoiesis, and thus may have a protective role against lymphoma [21]. Data regarding the influence of estrogen on lymphoma are conflicting, with retrospective studies showing a protective role against lymphoma [22], however, a large prospective study showed no difference in the incidence of lymphoma regarding estrogen use [23]. More interesting data have emerged in recent years regarding estrogen receptor beta (ERβ) in diffuse large B cell lymphoma (DLBCL). ERβ is significantly more expressed on DLBCL cells in female as well as male patients and estrogen exposure of DLBCL cell lines was shown to be protective against apoptosis. These findings suggest a potential role for ERβ antagonist tamoxifen in the treatment of lymphomas [24].

One limitation of this case is the persistence of positive end-of-treatment PET-CT in patients with primary mediastinal B cell lymphomas, while only 20% of these patients represent treatment failure. In these patients, serial PET-CT scans allow for identification of refractory patients which show an increase in the SUV of persistent malignancy, as is the case in the patient described, whereas in patients with persistent positive PET-CT but with remission of disease, masses show decreasing SUV [25].

## 5. Conclusions

Further research regarding characterization of FMCs, differentiation, expression profile and tumor infiltration is needed in order to establish a causality of events and support the hypothesis raised by this clinical observation. The potential clinical applications of microchimerism principles, however, warrant a deeper analysis of such cases which may offer potential clinical insights towards unraveling the mechanisms behind FMCs’ influence on cancer.

## Data Availability

All data is available upon reasonable request.

## Abbreviations

SR, spontaneous remission; FMCs, fetal microchimeric cells; CT, computer tomography; LDH, lactate dehydrogenase; SpO2, peripheral O2 saturation; INR, international normalized ratio; Hb, hemoglobin; CD, cluster of differentiation; R-DA-EPOCH, rituximab, dose-adjusted etoposide, prednisone, vincristine, cyclophosphamide and doxorubicin; R-CHOEP, rituximab, cyclophosphamide, doxorubicin, vincristine, prednisone, etoposide; PET-CT, positron emission tomography–computer tomography; SUV, standardized uptake value; R-ICE, rituximab, ifosfamide, carboplatin; DNA, deoxyribonucleic acid; gDNA, genomic DNA; RT PCR, reverse transcription polymerase chain reaction; CAR-T, chimeric antigen receptor T cell; GVL, graft versus leukemia; MST, micro stem cell transplantation.
